# Changes of serum calcium levels after thyroid surgery in the elderly

**DOI:** 10.1186/1471-2318-11-S1-A41

**Published:** 2011-08-24

**Authors:** G Patrizi, S Federici, M Fazi, F Pelle, L Fiengo, L Venturini, G Di Rocco, D Giannotti, F Frezzotti, R Giordano, A Redler

**Affiliations:** 1 Dipartimento di Scienze Chirurgiche, Policlinico Umberto I, Università di Roma“La Sapienza”, Rome, Italy

## Background

The reasons of transient hypocalcemia in the elderly, frequent after thyroid surgery, were investigated.

## Materials and methods

Serum total calcium and phosphoremia were determined in all patients who underwent thyroidectomy before and after surgery. Beside these, total protein, albumin and parathormone levels were also determined. The daily changes in serum calcemia and protein levels were measured in these patients.

## Results

Transient, mild but significant decrease of serum calcium levels was observed after surgery in up to a fifth of patients, while severe hypocalcemia was shown in about 5% of patients. The reduction of calcemia was more evident in elderly patients, with a concomitant reduction of albuminemia. In severe hypocalcemia, in addition to these two findings, decrease of parathormone levels was shown.

## Conclusions

We discuss the reasons for these findings, which are more frequently observed in elderly patients and the possible clinical implications to advise them to undergo total thyroidectomy.

